# Complete genome sequences of 12 bacterial strains from the honey bee gut, resolved with long-read nanopore sequencing

**DOI:** 10.1128/mra.00775-23

**Published:** 2024-01-09

**Authors:** Samuel A. Acheampong, Muhammet Fatih Polat, Waldan K. Kwong

**Affiliations:** 1 Instituto Gulbenkian de Ciência, Rua da Quinta Grande, Oeiras, Portugal; Indiana University, Bloomington, Bloomington, Indiana, USA

**Keywords:** microbiome, gut symbiont, insects, *Snodgrassella*, *Gilliamella*, *Serratia*, *Lactobacillus*, *Bifidobacterium*, *Bartonella*

## Abstract

We report the *de novo* sequencing of six bacterial strains isolated from the Western honey bee, as well as the resequencing of six strains that have existing draft genomes, to obtain complete, chromosomal-level assemblies. These strains include the bee gut symbiont genera *Bartonella, Bifidobacterium, Snodgrassella, Gilliamella, Lactobacillus*, and the opportunistic pathogen *Serratia marcescens* KZ11.

## ANNOUNCEMENT

Social bees harbor communities of specialized, symbiotic gut bacteria (1). There are currently >600 sequenced genomes of bee gut symbionts, and these genomes have played a crucial role in revealing how the bee gut community forms and how the symbionts benefit their host (2–4). However, few of these genomes are complete, which poses a challenge for the study of genome organization and evolution. Here, we use nanopore-based sequencing to obtain closed genomes for 12 bacterial strains isolated from the Western honey bee *Apis mellifera*, including 6 previously characterized strains for which only Illumina-based draft genomes exist.

Bacterial strains were grown at 35°C and 5% CO_2_ in deMan, Rogosa, and Sharpe (MRS) broth (*Bifidobacterium* and *Lactobacillus* spp.), tryptic soy broth (*Serratia* and *Snodgrassella*), or on tryptic soy agar with 5% sheep’s blood (*Bartonella* and *Gilliamella* spp.), after which total DNA was extracted as previously described (1). Oxford Nanopore Technologies (ONT) genomic sequencing libraries were prepared with the Native Barcoding Kit 96 V14 and Ligation Sequencing Kit V14 without shearing or size selection and sequenced on the ONT MinION platform using R10.4.1 flow cells (5). Base calling was performed with Guppy 6.4.6+ae70e8f using the template r10.4.1_e8.2_260bps_sup.jsn. Adapter and barcode trimming was enabled, and minimal qscore was adjusted to 10. Nanopore reads were assembled with Flye 2.9.2 (6), which produced high-coverage (>130×) closed, circular chromosomal assemblies for 10 of the 12 strains (Table 1). For all software, default parameters were used except where noted. Strains K-FP5 and wkB9, K-FP18 and ESL0169, and K-MP18 and wkB339 were pooled before nanopore barcoding and sequencing. In these cases, the --meta option in Flye was used during assembly.

**TABLE 1:** Genome assembly statistics and metadata for the sequenced strains

	Species	*Bartonella apihabitans*	*Bifidobacterium asteroides*	*Gilliamella apicola*	*Gilliamella apis*	*Gilliamella apis*	*Gilliamella apis*	*Lactobacillus apis*	*Lactobacillus helsingborgensis*	*Serratia marcescens*	*Snodgrassella alvi*	*Snodgrassella alvi*	*Snodgrassella alvi*
	**Strain**	K-FP28	K-FP5	ESL0178	ESL0169	ESL0172	K-MP18	K-MP7	K-FP18	KZ11	K-FP26	wkB9	wkB339
	**Source**	this study	this study	(7)	(7)	(7)	this study	this study	this study	(8)	this study	(9)	(9)
	**Host**	*Apis mellifera iberiensis*	*Apis mellifera iberiensis*	*Apis mellifera*	*Apis mellifera*	*Apis mellifera*	*Apis mellifera iberiensis*	*Apis mellifera iberiensis*	*Apis mellifera iberiensis*	*Apis mellifera*	*Apis mellifera iberiensis*	*Apis mellifera*	*Apis mellifera*
	**Location**	Lisbon, Portugal	Lisbon, Portugal	Lausanne, Switzerland	Lausanne, Switzerland	Lausanne, Switzerland	Lisbon, Portugal	Lisbon, Portugal	Lisbon, Portugal	Austin, TX, USA	Lisbon, Portugal	New Haven, CT, USA	Genting, Malaysia
	**Collection date (YYYY-MM-DD)**	2022–09-15	2022–09-15	2015–01-01	2015–11-01	2014–12-01	2022–11-07	2022–11-07	2022–09-15	2016–04	2022–09-15	2011–05-25	2014–07-27
	**NCBI BioSample accession**	SAMN36908620	SAMN36908621	SAMN36908625	SAMN36908623	SAMN36908624	SAMN36908622	SAMN36908627	SAMN36908626	SAMN36908628	SAMN36908629	SAMN36908630	SAMN36908631
	**NCBI SRA accession**	SRR25584835	SRR25584834	SRR25584830	SRR25584832	SRR25584831	SRR25584833	SRR25584829, SRR25584826	SRR25584832	SRR25584828	SRR25584827	SRR25584834	SRR25584833
	**NCBI Genome accession**	CP132387, CP132386, CP132385	CP132384	CP132380	CP132382	CP132381	CP132383	CP132378	CP132379	CP132377	CP132376	CP132375	CP132374
	**NCBI Assembly accession**	ASM3075875v1	ASM3075877v1	ASM3075859v1	ASM3075863v1	ASM3075861v1	ASM3075873v1	ASM3075857v1	ASM3075867v1	ASM3075855v1	ASM3075869v1	ASM3075865v1	ASM3075871v1
	**Assembly size**	2,702,659 bp chromosome; 31,560 bp plasmid 1; 5,266 bp plasmid 2	2,083,947 bp chromosome	2,903,879 bp chromosome	2,447,942 bp chromosome	2,714,786 bp chromosome	2,633,180 bp chromosome	1,556,433 bp chromosome	1,929,403 bp chromosome	5,075,014 bp chromosome	2,406,612 bp chromosome	2,527,983 bp chromosome	2,491,841 bp chromosome
	**Coverage**	1433×	144×	435×	1171×	409×	230×	1.95× ONT; 433× Illumina	289×	382×	252×	136×	220×
	**Read number**	793,469	165,575	24,996	781,983	121,100	433,460	2,501 ONT; 3,238,112 Illumina	781,983	392,202	1,178,953	165,575	433,460
	**Read N50**	7,236	5,574	18,855	8,072	17,085	8,876	3,665 ONT	8,072	9,935	4,730	5,574	8,876
	**CheckM completeness (Bacteria; Lineage)**	98.28%; 98.26%	100%; 97.64%	100%; 98.87%	100%; 98.31%	100%; 98.87%	100%; 98.31%	98.28%; 97.32%	98.28%; 97.72%	96.91%; 99.74%	100%; 99.57%	100%; 99.57%	100%; 99.57%
	**CheckM contamination (Bacteria; Lineage)**	0%; 0%	0%; 0.87%	1.72%; 0.56%	1.72%; 0.56%	1.72%; 0.56%	1.72%; 0.56%	1.72%; 0.76%	1.72%; 1.78%	0%; 0.15%	1.72%; 1.28%	1.72%; 0.85%	1.72%; 0.85%
	**GC content**	45.40%	60.80%	34.20%	34.30%	33.60%	34.80%	37.00%	36.70%	59.70%	41.20%	41.30%	41.20%
	**Total genes**	2482	1700	2624	2207	2492	2384	1489	1818	4864	2107	2294	2222
	**Protein CDS**	2178	1614	2523	2130	2404	2306	1408	1703	4661	2017	2204	2126
	**rRNA copies**	2	2	4	4	4	4	4	4	7	4	4	4
	**Pseudogenes (frame-shifted/total)**	206/246	20/32	8/33	1/10	9/21	4/13	3/8	36/44	57/72	5/14	5/14	7/20
**Comparison to closest sequenced strain**	**Hit strain**	*Bartonella sp.* M0177	*Bifidobacterium asteroides* Bin2	*Gilliamella apicola* ESL0178	*Gilliamella apis* ESL0169	*Gilliamella apis* ESL0172	*Gilliamella* sp. W8126	*Lactobacillus apis* Hma11	*Lactobacillus helsingborgensis* IBH003	*Serratia marcescens* KZ11	*Snodgrassella* sp. ESL0304	*Snodgrassella* alvi wkB9	*Snodgrassella alvi* wkB339
	**Hit assembly**	ASM1610228v1	ASM96718v1	ASM320281v1	ASM320287v1	ASM320277v1	ASM1610218v1	ASM97073v1	ASM2642823v1	ASM291544v1	ASM1334499v1	ASM277773v1	ASM277781v1
	**Number of contigs in hit**	3	18	18	13	17	20	16	5	21	23	15	27
	**ANI to hit**	97.20%	96.75%	99.98%	100%	99.99%	97.82%	97.67%	97.49%	100%	98.02%	99.99%	99.99%

For *Snodgrassella alvi* wkB339, two gaps were closed by manually inspecting the mapping of raw reads to contigs using Geneious Prime 2023.0.4 (Biomatters Ltd.) and the Minimap2 2.2.0 plugin (10). For *Lactobacillus apis* K-MP7, the nanopore-based assembly was incomplete (1.95× coverage); therefore, we additionally generated a short-read library from the same DNA sample using the Nextera XT DNA Kit for 2 × 150 bp sequencing on the NextSeq 2000 platform with P2 reagents (Illumina Inc.). Reads were quality controlled using AfterQC 0.9.7 (11) with the options --n_base_limit = 1, --seq_len_req = 50. A hybrid assembly using nanopore and Illumina reads was made with SPAdes 3.15.5 (12), and the *L. apis* K-MP7 genome was closed through several rounds of polishing with Pilon 1.23 (13). To check for potential misassemblies and genomic rearrangements, the Mauve 1.1.3 (14) plugin in Geneious Prime was used to align assemblies against complete genomes of the same species, if available, from the National Center for Biotechnology Information Reference Sequence (NCBI RefSeq) database. CheckM 1.2.2 (15) was run to determine assembly completeness and contamination, using both taxon_set and lineage_set workflows.

Finished chromosomes were reordered such that the *dnaA* gene was placed at the start of the sequence. Genome annotation was carried out using the NCBI Prokaryotic Genome Automatic Annotation Pipeline 6.6 (16). Based on average nucleotide identity (ANI) (17), our new strains’ genomes were 96.7%–98.0% similar to that of the closest hits in NCBI RefSeq (Table 1). For the six resequenced strains, we found marked improvements in assembly contiguity, coverage, and total assembly size.

## Data Availability

All data associated with these genomes are deposited under NCBI BioProject number PRJNA1003869. See Table 1 for strain specific accessions.

## References

[1] Kwong WK, Medina LA, Koch H, Sing K-W, Soh EJY, Ascher JS, Jaffé R, Moran NA. 2017. Dynamic microbiome evolution in social bees. Sci Adv 3:e1600513. doi:10.1126/sciadv.1600513 28435856 PMC5371421

[2] Powell JE, Leonard SP, Kwong WK, Engel P, Moran NA. 2016. Genome-wide screen identifies host colonization determinants in a bacterial gut symbiont. Proc Natl Acad Sci U S A 113:13887–13892. doi:10.1073/pnas.1610856113 27849596 PMC5137728

[3] Raymann K, Moran NA. 2018. The role of the gut microbiome in health and disease of adult honey bee workers. Curr Opin Insect Sci 26:97–104. doi:10.1016/j.cois.2018.02.012 29764668 PMC6010230

[4] Bonilla-Rosso G, Engel P. 2018. Functional roles and metabolic niches in the honey bee gut microbiota. Curr Opin Microbiol 43:69–76. doi:10.1016/j.mib.2017.12.009 29309997

[5] Sereika M, Kirkegaard RH, Karst SM, Michaelsen TY, Sørensen EA, Wollenberg RD, Albertsen M. 2022. Oxford nanopore R10.4 long-read sequencing enables the generation of near-finished bacterial genomes from pure cultures and metagenomes without short-read or reference polishing. Nat Methods 19:823–826. doi:10.1038/s41592-022-01539-7 35789207 PMC9262707

[6] Kolmogorov M, Bickhart DM, Behsaz B, Gurevich A, Rayko M, Shin SB, Kuhn K, Yuan J, Polevikov E, Smith TPL, Pevzner PA. 2020. metaFlye: scalable long-read metagenome assembly using repeat graphs. Nat Methods 17:1103–1110. doi:10.1038/s41592-020-00971-x 33020656 PMC10699202

[7] Ellegaard KM, Engel P. 2018. New reference genome sequences for 17 bacterial strains of the honey bee gut microbiota. Microbiol Resour Announc 7:e00834-18. doi:10.1128/MRA.00834-18 30533872 PMC6211356

[8] Raymann K, Shaffer Z, Moran NA. 2017. Antibiotic exposure perturbs the gut microbiota and elevates mortality in honeybees. PLoS Biol 15:e2001861. doi:10.1371/journal.pbio.2001861 28291793 PMC5349420

[9] Steele MI, Kwong WK, Whiteley M, Moran NA. 2017. Diversification of type VI secretion system toxins reveals ancient antagonism among bee gut microbes. mBio 8:e01630-17. doi:10.1128/mBio.01630-17 29233893 PMC5727410

[10] Li H. 2018. Minimap2: pairwise alignment for nucleotide sequences. Bioinformatics 34:3094–3100. doi:10.1093/bioinformatics/bty191 29750242 PMC6137996

[11] Chen S, Huang T, Zhou Y, Han Y, Xu M, Gu J. 2017. AfterQC: automatic filtering, trimming, error removing and quality control for fastq data. BMC Bioinformatics 18:80. doi:10.1186/s12859-017-1469-3 28361673 PMC5374548

[12] Prjibelski A, Antipov D, Meleshko D, Lapidus A, Korobeynikov A. 2020. Using spades de novo assembler. Curr Protoc Bioinformatics 70:e102. doi:10.1002/cpbi.102 32559359

[13] Walker BJ, Abeel T, Shea T, Priest M, Abouelliel A, Sakthikumar S, Cuomo CA, Zeng Q, Wortman J, Young SK, Earl AM. 2014. Pilon: an integrated tool for comprehensive microbial variant detection and genome assembly improvement. PLoS One 9:e112963. doi:10.1371/journal.pone.0112963 25409509 PMC4237348

[14] Darling ACE, Mau B, Blattner FR, Perna NT. 2004. Mauve: multiple alignment of conserved genomic sequence with rearrangements. Genome Res 14:1394–1403. doi:10.1101/gr.2289704 15231754 PMC442156

[15] Parks DH, Imelfort M, Skennerton CT, Hugenholtz P, Tyson GW. 2015. CheckM: assessing the quality of microbial genomes recovered from isolates, single cells, and metagenomes. Genome Res 25:1043–1055. doi:10.1101/gr.186072.114 25977477 PMC4484387

[16] Tatusova T, DiCuccio M, Badretdin A, Chetvernin V, Nawrocki EP, Zaslavsky L, Lomsadze A, Pruitt KD, Borodovsky M, Ostell J. 2016. NCBI prokaryotic genome annotation pipeline. Nucleic Acids Res 44:6614–6624. doi:10.1093/nar/gkw569 27342282 PMC5001611

[17] Goris J, Konstantinidis KT, Klappenbach JA, Coenye T, Vandamme P, Tiedje JM. 2007. DNA-DNA hybridization values and their relationship to whole-genome sequence similarities. Int J Syst Evol Microbiol 57:81–91. doi:10.1099/ijs.0.64483-0 17220447

